# Synergistic Mechanisms and Product Regulation in the Co-Pyrolysis of Biomass and Food Packaging Waste: A Study Based on Reaction Kinetics and GHG Calculation

**DOI:** 10.3390/foods15061098

**Published:** 2026-03-20

**Authors:** Gang Li, Xingyang Lai, Jue Gong, Tong Zhang, Ke Xu, Zhengyang Feng, Xiaolong Yao

**Affiliations:** 1 School of Computer and Artificial Intelligence, Beijing Technology and Business University, Beijing 100048, China; ligang@btbu.edu.cn (G.L.); laixingyang1226@163.com (X.L.); 2431062201@st.btbu.edu.cn (J.G.); ztong000608@163.com (T.Z.); 2330602111@st.btbu.edu.cn (K.X.); 2 Department of Environmental Science and Engineering, Beijing Technology and Business University, Beijing 100048, China; 18753484312@163.com

**Keywords:** co-pyrolysis, food waste valorization, circular economy, synergistic effect, GHG calculation

## Abstract

To address the mounting environmental burden caused by solid waste from the food supply chain—specifically agricultural residues and plastic packaging—this study systematically investigated the synergistic mechanisms and product regulation pathways in the co-pyrolysis of four representative food processing by-products—rice husk, pine wood, corn stover, and chestnut shell—with polypropylene, a common food packaging material. A comprehensive methodology integrating thermogravimetric analysis, kinetic modeling, and product characterization was employed. The results demonstrate that incorporating polypropylene into co-pyrolysis systems, such as those involving waste oil, significantly reduces the average activation energy, indicating a catalytic effect that enhances reaction kinetics. Notably, the co-catalytic interaction between corn stover and PP led to a substantial 54.90% reduction in oxygen content, underscoring PP’s role as an effective hydrogen donor that promotes deoxygenation and free radical reactions, thereby increasing hydrocarbon production. At an optimal pyrolysis temperature of 600 °C, product distribution was effectively regulated: the hydrocarbon yield in the CP (corn stover/PP) system increased from 39.8% to a maximum of 65.6%, reflecting a targeted conversion of oxygenated compounds into high-value hydrocarbons. Furthermore, greenhouse gas (GHG) emission calculation and techno-economic analyses indicate that a natural gas-assisted co-pyrolysis process (Scenario C) can generate a net daily profit of 1835 RMB while reducing annual CO_2_ emissions by 6515 tons, demonstrating both economic feasibility and environmental benefits. This study provides a theoretical foundation for the circular economy in the food industry, offering a viable technical pathway for the simultaneous treatment of organic food waste and packaging plastics, thereby supporting the sustainable development of the agri-food sector.

## 1. Introduction

With the rapid expansion of the global food industry, the management of solid waste generated along the supply chain has become a critical environmental challenge [1]. Vast quantities of lignocellulosic agricultural residues (such as rice husks, corn stover, and fruit shells) and non-biodegradable food packaging materials (primarily plastics like polypropylene) are discarded annually, posing severe threats to ecosystems and human health if treated by conventional landfilling or open burning [2]. Consequently, developing sustainable technologies for the resource recovery of these agri-food wastes is an urgent priority for the circular bioeconomy [3]. Pyrolysis technology stands out as a promising thermochemical pathway for valorizing organic waste, capable of converting biomass into bio-oil, biochar, and syngas [4]. However, bio-oil derived solely from lignocellulosic biomass often suffers from high oxygen content and low heating value, limiting its direct application as a clean fuel [4]. Meanwhile, the accumulation of waste plastics from food packaging continues to exacerbate “white pollution” [5].

To address these challenges, biomass co-pyrolysis technology has been developed, which synergistically converts biomass with other hydrogen-rich feedstocks, enhancing the production of bio-oil, potentially enhancing the quality of the resulting pyrolysis oil [4,6,7]. A synergistic effect occurs when the combined outcome of interactions between two or more components surpasses the sum of their individual effects [8]. Studies have demonstrated that co-pyrolysis can significantly enhance product yields by leveraging synergies between various biomass sources, as seen in research examining the impact of different atmospheres and the use of diverse biomass feedstocks [6,7]. Diverse co-pyrolysis feedstocks, particularly waste plastics like polypropylene (PP), exhibit distinct advantages [9]. Brebu et al. investigated the co-pyrolysis of pine cones with polyethylene, PP, and polystyrene, finding that the interaction between the pine cones and polymers played a key role in product yields [10]. Ahmed et al. reported the synergistic effects of co-pyrolyzing biomass and polyethylene at different mass fractions, and the results demonstrated that the co-pyrolysis of biomass [11] and waste plastics mitigates the shortcomings of individual pyrolysis due to enhanced synergistic interactions. Research has demonstrated that co-pyrolysis of plastics with lignocellulosic or cellulosic biomass can increase the gas content and enhance the overall quality of the resulting biochar [9,12]. During the co-pyrolysis of lignocellulosic biomass and PP, the content of oxygenated compounds in the resulting oil decreases, and more than 20 types of aromatic compounds are produced [13]. PP serves as a hydrogen donor during the co-pyrolysis process with lignocellulose, promoting liquefaction while minimizing char deposition [14,15]. Economically, the co-pyrolysis of biomass with plastics has been shown to yield higher returns than the pyrolysis of biomass in isolation, as evidenced by studies on hydrogen production and the enhancement of bio-oil quality [9,12,16].

Despite the significant advancements in the field of biomass and plastic co-pyrolysis, as evidenced by the extensive research on methane pyrolysis, catalytic biomass pyrolysis, and the use of combined catalysts [17,18,19], there remain several critical scientific questions that require further investigation. First, the synergistic effects and interaction pathways between biomass components are crucial to understanding the overall process outcomes, as evidenced by studies that have explored the impact of these interactions on bio-oil yield, gas generation, and torrefaction severity [17,18,19]. For instance, cellulose, hemicellulose, lignin, and polypropylene are crucial for understanding their behavior in composite materials. Reactions involving hydrogen transfer and free radical coupling are complex and frequently lack detailed quantitative characterization. As demonstrated in studies, researchers have explored the intricacies of these steps, such as the radical relay strategy in olefin difunctionalization and the impact of quantum tunneling on hydrogen transfer reactions [20,21]. Second, the structure–activity relationship governing the change in activation energy during co-pyrolysis has not yet been established, making it difficult to predict the synergistic potential of different feedstock combinations. Additionally, the formation pathways of hydrocarbons and phenols are competitive, and there is a lack of strategies for targeted product regulation based on the combined adjustment of the three-component ratio and reaction temperature. To date, a comprehensive evaluation system that integrates process optimization with energy structure to thoroughly assess the economic viability and environmental impacts of co-pyrolysis processes has not been fully developed.

In light of this, the present study selected four representative biomass feedstocks—rice husk (representing high-ash agricultural residues), pine wood (representing lignocellulosic biomass), corn stalk (representing common straw-type biomass), and chestnut shell (representing high-lignin feedstock)—to systematically investigate their co-pyrolysis behavior with polypropylene. From the dual perspectives of reaction kinetics and greenhouse gas emission calculation, an integrated research framework encompassing “reaction mechanism–product regulation–industrial pathway” was established. The study aims to quantify synergistic effects during co-pyrolysis, elucidate the evolution of product distribution across temperature gradients, and evaluate the GHG reduction potential of the proposed co-pyrolysis pathway under a gate-to-gate system boundary, together with techno-economic analysis based on experimental data to support industrial scalability and decision-making. The findings are intended to bridge the gap between phenomenological observation and mechanistic understanding, as well as between laboratory-scale research and industrial applicability, thereby paving the way for novel technological pathways enabling the efficient transformation of waste into clean energy.

## 2. Materials and Methods

### 2.1. Feedstocks

The experimental feedstocks selected for this study comprise rice husk, pine wood, corn stover, chestnut shell, and PP (Mn = 5000, Mw = 12,000). The biomass materials were procured from a wood processing plant in the Fangshan District and agricultural land in the Miyun District of Beijing, China. The PP sample was provided by Aladdin, a chemical reagent manufacturer. All feedstocks are subjected to strict pre-treatment prior to use: initially cleaned thoroughly to remove impurities, subsequently dried in an oven at 60 °C for 24 h to ensure complete moisture removal, and finally ground and sieved through a 50-mesh sieve to achieve uniform particle size distribution.

The processed rice husk, pine wood, corn stover, and chestnut shell were thoroughly mixed with PP in a 1:1 mass ratio. The mixtures were labeled as RP (Rice husk–PP), PIP (Pine wood–PP), CP (Corn stover–PP), and CTP (Chestnut shell–PP) and were respectively stored in sealed containers for subsequent experiments.

### 2.2. Thermogravimetric Analysis

Thermogravimetric analysis (TGA) was conducted using an SDT Q600 thermal analyzer (TA Instruments, New Castle, IN, USA), which is equipped with a dual-arm parallel balance design and offers a temperature range from room temperature to 1500 °C, ensuring precise measurements. For each experiment, approximately 15 mg of a biomass sample (such as rice husk, pine wood, corn stover, or chestnut shell) or its mixture with PP was precisely weighed and placed into the instrument’s crucible. The pretreated samples were dried at 105 °C until the mass stabilized, and the recorded percentage of mass loss was recorded as the moisture content on an air-dried basis. The sample was then heated to 900 °C for 7 min under a nitrogen (N_2_) atmosphere for pyrolysis. During the experiment, N_2_ gas was continuously blown at a rate of 20 mL/min to maintain the inert atmosphere required for pyrolysis. The mass loss rate was measured, and the air-dried volatile matter content was calculated after subtracting the M_ad_ (mass loss due to dehydration). The sample was then ashed at 550 °C until the mass remained constant, and the mass percentage of the residue was determined as the ash content on an air-dried basis.

### 2.3. Pyrolysis Kinetics

The pyrolysis process of biomass can be described by Equation (1):

(1) $$d\alpha /dt=K\left(t\right)f\left(\alpha \right)$$

where *α* represents the conversion rate, and *t* denotes the temperature.

The Arrhenius equation is frequently employed to describe the relationship between the reaction rate constant and temperature, as illustrated in Equation (2):

(2) $$k=\alpha \cdot exp^{-\frac{E}{RT}}$$

where *A* is the pre-exponential factor; activation energy (*E*) is the minimum energy required for reactant molecules to transform into activated molecules, measured in kJ/mol. The universal gas constant (*R*), also known as the ideal gas constant, is 8.314 × 10^−3^ kJ/mol/K, and the thermodynamic temperature (*T*) is measured in Kelvin (*K*).

The conversion rate α is defined as [22]:

(3) $$\alpha =\frac{m_{0}-m_{t}}{m_{0}-m_{f}}$$

In the context of thermal analysis, m_0_ represents the initial sample mass in milligrams (mg), *m_t_* is the sample mass at a given time *t* (mg), and *m_f_* denotes the final mass of the sample after pyrolysis (mg).

By substituting Equation (2) into Equation (1), we obtain Equation (4):

(4) $$d\alpha /dt=Aexp^{-\frac{E}{RT}}\cdot f\left(\alpha \right)$$

*β* is the heating rate:

(5) β=dT/dt

This ultimately yields Equation (6):

(6) $$\beta \left(d\alpha /dT\right)=Aexp^{-\frac{E}{RT}}\cdot f\left(\alpha \right)$$

#### 2.3.1. Kissinger–Akahira–Sunose (KAS) Kinetic Model

The KAS kinetic method is expressed by Equation (7) [23]:

(7) $$\ln\left(\beta /T^{2}\right)=\ln\left(AR/E_{\alpha }g\left(\alpha \right)\right)-E_{\alpha }/RT_{\alpha }$$

The overall reaction model can be represented by g(*α*). By applying linear regression to plot ln(*β*/*T*^2^) against 1/*T*, a straight line is obtained, from which the activation energy can be calculated based on the slope.

#### 2.3.2. Starink Kinetic Model

The Starink kinetic model is expressed by Equation (8) [24]:

(8) $$\ln\left(\beta /T^{1.92}\right)=Const.-1.0008\left(E_{\alpha }/RT_{\alpha }\right)$$

By plotting ln *β*/*T*^1.92^ against 1/*T*, a linear relationship is observed, from which the activation energy can be determined by evaluating the slope of the line.

### 2.4. Analysis of Pyrolysis Products

Pyrolysis experiments were conducted in a programmable pyrolysis unit. The experimental setup was set to heat at a rate of 10 °C/min and maintain the final temperature for 20 s. Initially, preliminary pyrolysis experiments were performed on individual samples of rice husk (RH), pine wood (PI), corn stover (CS), chestnut shell (CT), and polypropylene (PP), which were tested over a temperature range of 300 °C to 700 °C, with an experimental point set every 100 °C, as detailed in a study on rice husk-reinforced polypropylene composites. Subsequently, co-pyrolysis experiments were performed on combinations of RH, PI, CS, and CT with PP, with all mixtures were prepared at a 1:1 mass ratio.

A desktop scanning electron microscope (Phenom-World BV, Phenom XL, Eindhoven, The Netherlands) was used for analysis. The biochar samples obtained from the experiments were first coated with gold via sputtering to enhance conductivity and then imaged using a Scanning Electron Microscope (SEM) at 1500 magnification under a vacuum of 10^−5^ Pa. The SEM utilized an acceleration voltage of 22.00 kV to capture detailed surface morphology and elemental composition.

The gas and liquid components of the pyrolysis products were analyzed by a gas chromatography-mass spectrometry (GC-MS) system. Manual sample introduction was performed under He atmosphere with a sample volume of 0.2 mg. During the experiment, He gas was continuously blown at a rate of 5 mL/min to maintain the inert atmosphere required for pyrolysis. The gas chromatography analysis was performed using an Agilent HP-5 capillary column (Agilent Technologies, Santa Clara, CA, USA), known for its non-polar (5%-phenyl)-methylpolysiloxane stationary phase, which is widely used in gas chromatography for the separation and quantification of medium- to high-polarity substances. The column had a length of 30 m, an internal diameter of 0.25 mm, and a film thickness of 0.25 µm, with a split ratio set at 80:1. The temperature program began at an initial temperature of 40 °C and was maintained for 2 min. It then increased to 180 °C at a rate of 5 °C/min, and finally rose to 280 °C at a rate of 10 °C/min, holding at this temperature for 10 min. The mass spectrometry analysis was conducted over a scan range of *m*/*z* 35–450. The acquired mass spectra were compared with the NIST 17 mass spectral library to ascertain the qualitative characteristics of the compounds, as demonstrated in the examples, where mass spectral data was used to determine molecular formulas.

### 2.5. GHG Emission Calculation

The system boundary for the GHG calculation was defined as “gate-to-gate”, which commenced with the arrival of raw materials (biomass, waste plastics) at the factory gate and concluded with the completion of the pyrolysis process, along with the separation and collection of the three-phase products (oil, char, gas). This means the study did not consider the environmental burdens from the upstream collection and transportation of raw materials, nor the downstream refining, upgrading, or combustion of the products.

To assess the industrial viability of the co-pyrolysis technology, three industrial scenarios were formulated based on the system boundary. Scenario A illustrates the existing biomass pyrolysis operations at a collaborating company, featuring an annual processing capacity of 6500 tons. Scenario B was established as the baseline scenario, which represents a direct scale-up from laboratory-scale all-electric heating conditions and reflects the technology’s theoretical potential. Scenario C serves as an engineering optimization case, employing laboratory technology while integrating a hybrid power supply of electricity and natural gas to simulate real-world production conditions. Based on these scenarios, Scenarios A and C were comparatively evaluated. The key parameters such as product yields (oil, char, gas) and reaction conditions were sourced from the laboratory data of this study, with reasonable scale-up correction factors applied based on industrial experience [25,26,27]. The background data, encompassing the system’s processing scale, unit energy consumption (electricity), and utility data, were substantiated by the actual annual operational data from the partner enterprise’s existing facilities. The remaining solid waste is processed by a specialized facility at a cost of 1233 RMB per ton.

The Database (CLCD) indicates that the CO_2_ emission factor for the Chinese power grid has exhibited a declining trend, decreasing from 763.94 g/(kW·h) to 557.73 g/(kW·h) in 2021, which corresponds to a reduction of 27.0% [28]. This study focuses on GHG emissions expressed as CO_2_-equivalent. The methodology employed in this calculation is derived from the Fifth Assessment Report of the Intergovernmental Panel on Climate Change (IPCC), incorporating characteristic factors over a 100-year time horizon to quantify the processing of 1 metric ton of blended feedstock (biomass and PP in a 1:1 mass ratio).

Based on the system boundary of “gate-to-gate” and the actual data generated by the enterprise, the material and energy input–output data for the three scenarios were compiled, and the material and energy flows under three scenarios were quantified. Table 1 summarizes the key inputs and outputs.

## 3. Results and Discussion

### 3.1. Feedstock Analysis

Table 2 presents the results of elemental, proximate, and chemical composition analyses for the four types of biomass and PP. The elemental analysis data show that none of the biomass or PP samples contain sulfur. In the samples, RH exhibits the highest oxygen content at 55.70%, whereas CT has the lowest at 16.65%. It is generally believed that lower oxygen content in the feedstock is beneficial for improving the quality of bio-oil [29]. PP is abundant in hydrogen, with a content of 12.53%, suggesting its potential to act as a hydrogen donor during co-pyrolysis and provide an additional hydrogen source for the biomass [30]. Furthermore, the low fixed carbon content in PP indicates its limited potential as a fuel source, since fixed carbon plays a crucial role in generating heat during combustion [16,31]. However, its high volatile matter content (up to 99.29%) suggests a significant advantage in the pyrolysis process, as it can effectively lower the reaction temperature of the pyrolysis system, accelerate the reaction rate, and increase the added value of the products [32].

### 3.2. Co-Pyrolysis Analysis of Materials and PP

#### 3.2.1. Pyrolysis Characteristics of Biomass and PP

**Figure 1:**
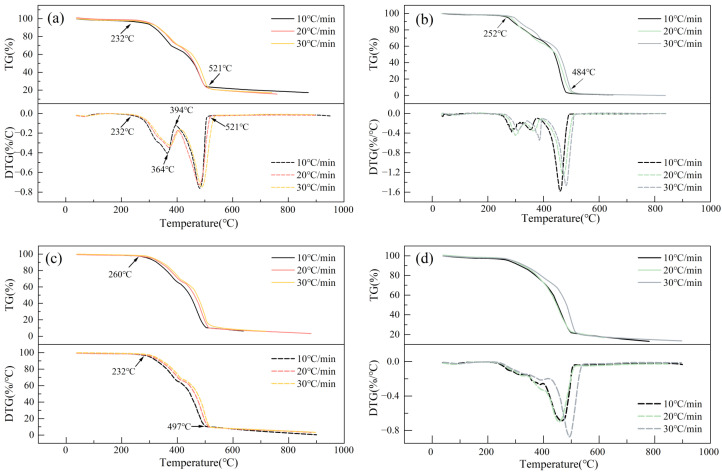
The TG-DTG curves of RP co-pyrolysis (**a**), CP co-pyrolysis (**b**), PIP co-pyrolysis (**c**), and CTP co-pyrolysis (**d**).

As shown by the TG/DTG curves in Figure 1, the co-pyrolysis processes of PP and different biomass blends exhibit distinct characteristics, as evidenced by the increased release of volatiles and changes in the pyrolysis kinetics, which are influenced by the biomass type and blending ratio. The co-processing of the four biomass types with PP exhibits common synergistic effects, while simultaneously displaying distinct characteristics due to differences in their chemical compositions. Generally, the TG/DTG curves for all co-pyrolysis systems follow a similar trend. Firstly, the DTG curves consistently display two primary mass loss peaks: the first, in the lower temperature range (approx. 300–400 °C), is primarily attributed to the decomposition of hemicellulose and cellulose in the biomass [33]; the second, in the higher temperature range (approx. 450–500 °C), is where the presence of lignin promotes the decomposition of PP [34]. Secondly, the co-pyrolysis of lignin and PP shows a significant synergistic effect, as demonstrated by a substantial increase in the yield of phenolic compounds and the lowest radical concentration in the bio-oil obtained from lignin/PP [35,36]. The addition of PP facilitates the secondary pyrolysis reaction of cellulose, while the secondary pyrolysis of lignin is inhibited, resulting in a substantial reduction in the final residual mass of all co-pyrolysis systems [37,38]. Additionally, as the heating rate increases from 10 °C/min to 30 °C/min, the TG and DTG curves of all samples shift towards higher temperatures, exhibiting a typical thermal hysteresis phenomenon [39,40,41].

However, notable differences exist in the specific co-pyrolysis behaviors among different biomass systems. The initial decomposition temperatures vary, with RP having the lowest (approx. 232 °C) while the PIP system operates at the highest temperature (approximately 260 °C), which may be influenced by the content and composition of volatile matter inherent in each type of biomass. Notable differences are also observed in the degree of decomposition. The complete reactions of CP and PIP, with final residual masses below 2%, suggest a highly efficient synergistic decomposition, as opposed to RP, which has a relatively high residual mass of approximately 18.1%, indicating a less efficient synergistic decomposition. In terms of DTG peak shapes, RP, CP, and PIP all show a typical double-peak structure. However, the CTP system is unique, with its DTG curve showing only a single, broad mass loss peak in the 400–500 °C range. This may be attributed to the highly overlapping decomposition temperature ranges of hemicellulose and cellulose in CT. Although the co-pyrolysis of biomass and PP follows a similar reaction framework, the chemical composition of the biomass itself (e.g., the content and ratio of cellulose, lignin) is a key intrinsic factor that governs the intensity of the synergistic effect, the decomposition temperature range, and the final product residue [15].

#### 3.2.2. Kinetic Analysis

Mathematical models play a key role in revealing chemical reaction mechanisms [42]. To investigate the reaction kinetics of the co-pyrolysis process involving four types of biomass and PP, this study employed two well-established analytical approaches. The iso-conversional methods, specifically the KAS and Starink methods, were applied to analyze TGA data obtained from multiple samples under varying heating rates. Both methods yielded highly linear correlations, with determination coefficients (R^2^) exceeding 0.95, thereby demonstrating their reliability and suitability for kinetic analysis within the scope of this study. It should be noted, however, that although the kinetic models exhibit strong fitting performance, further validation across a wider range of experimental conditions and biomass feedstocks—using robust techniques such as cross-validation and nested cross-validation—is required to enhance their predictive generalizability.

**Figure 2:**
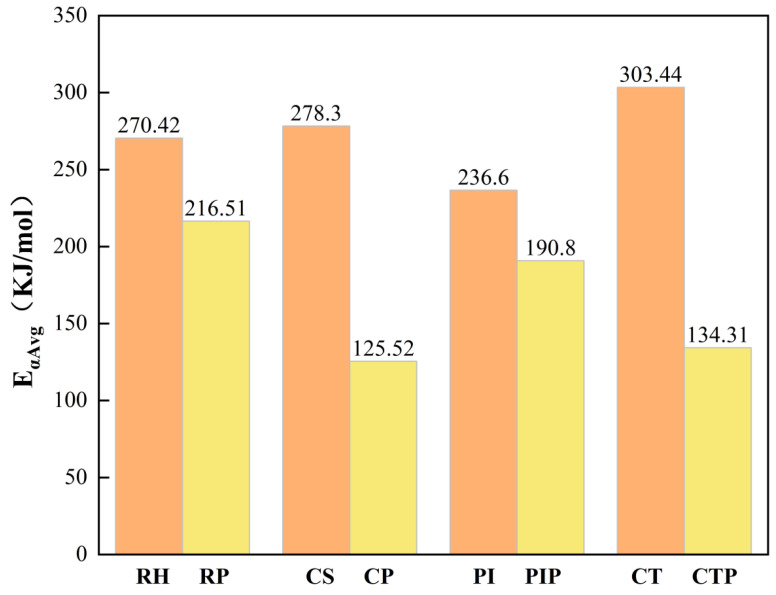
Average activation energy for individual and co-pyrolysis of biomass and PP, as studied in the synergistic pyrolysis of waste polypropylene and biomass.

During individual pyrolysis processes, CT demonstrated the highest reaction threshold, followed by CS, RH, and PI. The activation energy value for the individual pyrolysis of PP was largely consistent with the range reported in the literature [41], with minor differences likely attributable to variations in feedstock particle size across different studies. Generally, smaller particle sizes, due to their larger specific surface area, promote internal heat transfer, leading to lower activation energy and higher pyrolysis rates [43]. As illustrated in Figure 2, the activation energy for co-pyrolysis changed correspondingly following the addition of PP, with the values decreasing in the following order: RP > PIP > CTP > CP. The most significant reduction in activation energy (54.90%) was observed during the co-pyrolysis of corn stover (CS) with PP. This substantial decrease is attributed to the synergistic interaction between biomass and plastic, which enhances hydrogen transfer and catalytic effects, thereby lowering the energy barrier for thermal decomposition. A comparable 20% reduction in activation energy was reported in previous studies involving the co-pyrolysis of RH and PP, such as in investigations on the impact of torrefaction on rice husk pyrolysis [17]. These findings indicate that during the initial stage of co-pyrolysis, the incorporation of PP promotes favorable reaction kinetics through enhanced synergy. In the later stages, secondary reactions are increasingly influenced by the lignin component. The high efficiency of the cellulose–PP system in facilitating hydrogen transfer is further confirmed, providing a kinetic foundation for reactor design and optimization of temperature programming.

### 3.3. Study of Pyrolysis Products

#### 3.3.1. Morphological Evolution

**Figure 3:**
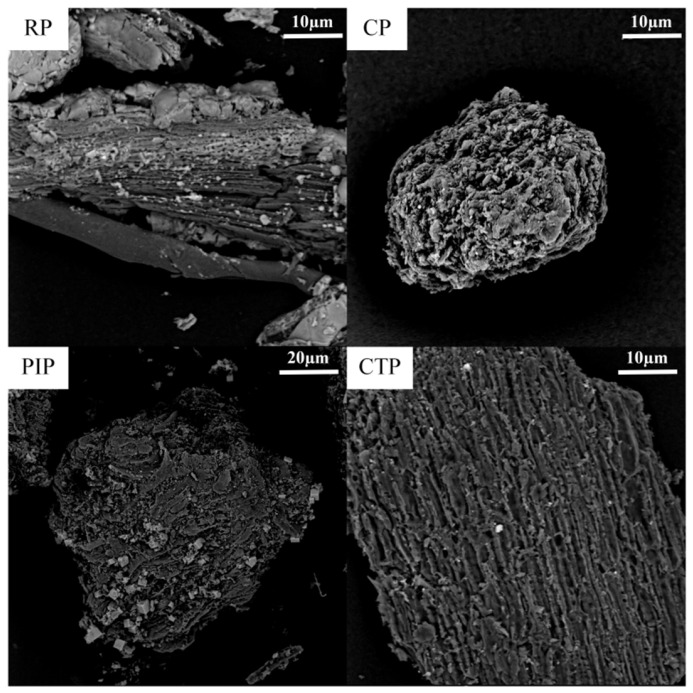
SEM of biochar derived from the co-pyrolysis of biomass and PP.

SEM images of biochar obtained from the co-pyrolysis of various biomass feedstocks with PP are presented in Figure 3. Distinct morphological differences are observed across the different biomass–PP combinations. Biochar derived from rice husk (RH) and PP exhibits a relatively rough, layered structure with limited porosity. In contrast, the biochar from corn stover (CS) and PP co-pyrolysis displays a more spherical, particle-like morphology with a smooth surface, indicating that volatile compounds released from CS may interact with PP decomposition products, promoting uniform char deposition. The biochar produced from pine wood (PI) and PP shows a higher degree of porosity and an uneven particle distribution, likely due to complex interactions between the high lignin content in PI and hydrocarbons generated during PP decomposition, which help preserve the porous architecture. Biochar from chestnut shell (CT) and PP co-pyrolysis exhibits a dense, fibrous structure with well-defined surface features, potentially resulting from the rapid decomposition of cellulose and hemicellulose in CT, which synergistically enhances the char formation process during co-pyrolysis with PP. These observations demonstrate that the chemical composition and structural characteristics of the biomass significantly influence the microstructural evolution of biochar during co-pyrolysis with PP, consistent with previous studies on biomass-derived pyrolysis behavior and biochar properties [44].

#### 3.3.2. Analysis of Co Pyrolysis Products

**Figure 4:**
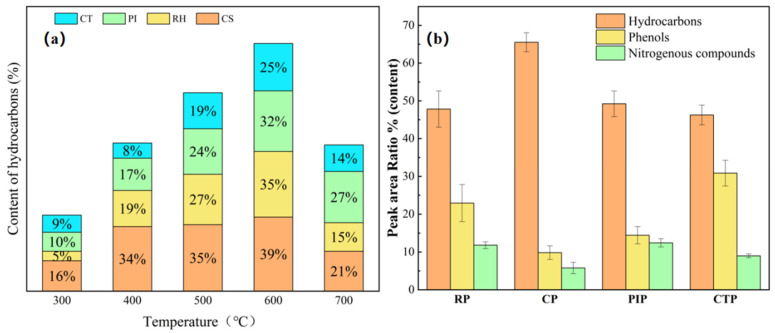
Change in hydrocarbon product yield at different pyrolysis temperatures (**a**); Product distribution from biomass and PP co-pyrolysis (**b**).

To maximize the production of hydrocarbon compounds, this study systematically investigated the influence of temperature on the distribution of co-pyrolysis products. As illustrated in Figure 4a, the total hydrocarbon yield across all co-pyrolysis systems exhibits a distinct temperature-dependent trend, characterized by an initial increase followed by a subsequent decline. In the lower temperature range of 300–400 °C, biomass decomposition is incomplete, with dehydration and decarboxylation being the predominant reaction pathways; consequently, hydrocarbon yields remain low. As the temperature rises to 500–600 °C, secondary cracking, deoxygenation, and synergistic interactions are significantly enhanced, leading to a marked increase in hydrocarbon formation, with peak yield observed at 600 °C [45]. However, further increasing the temperature to 700 °C promotes excessive cracking of the generated hydrocarbons, resulting in their conversion into lighter gaseous species and a consequent reduction in overall hydrocarbon yield [46]. A similar trend is observed for specific aromatic compounds such as benzene, whose yield also peaks at 600 °C. Therefore, 600 °C was determined to be the optimal pyrolysis temperature for subsequent detailed investigations.

At the optimal temperature of 600 °C, a detailed comparative analysis was conducted on the product distribution across the four co-pyrolysis systems (Figure 4b) to elucidate the influence of different biomass components on synergistic effects. Previous studies have demonstrated that the extent of synergistic interactions in biomass conversion processes is strongly dependent on its chemical composition, particularly evident in enhanced thermochemical interactions during co-pyrolysis. Among the tested biomass types, corn stover (CP), which is rich in cellulose and hemicellulose, exhibited the most pronounced synergistic effect, increasing the hydrocarbon yield from 39.8%—obtained from pyrolysis of corn stover alone—to 65.6%. This enhancement can be attributed to the abundant free radicals generated from polypropylene (PP) cracking at 600 °C, which effectively promote the deoxygenation and molecular rearrangement of volatiles derived from cellulose and hemicellulose, thereby maximizing hydrocarbon formation [47]. In contrast, CTP, with its highest lignin content, had the highest proportion of phenolic compounds in its product stream, while the increase in hydrocarbon yield was relatively limited [48,49,50]. This indicates that although alkyl free radicals generated during PP pyrolysis can abstract hydrogen atoms from the hydroxyl groups of biomass—resulting in the formation of alkanes and phenoxy free radicals—and thereby convert certain phenolic compounds into hydrocarbons, the high concentration of phenolic compounds derived from lignin decomposition remains a defining characteristic of the system [51]. The generation of hydroxyl free radicals during biomass pyrolysis, along with reactions involving phenoxy, catechol, and hydrogen free radicals originating from PP, collectively contribute to the sustained formation of phenols [52,53]. The synergistic effects observed in the RP and PIP systems fall within an intermediate range relative to those of CP and CTP, which corresponds to their respective cellulose/lignin ratios. Notably, given that PP contains no nitrogen, the proportion of nitrogenous compounds across all systems remained consistently stable at approximately 10%, indicating no significant influence on the overall composition.

In summary, external factors such as temperature and biomass type play a critical role in determining hydrocarbon production during the co-pyrolysis of biomass and PP. Internally, the relative proportions of the three primary biomass components—cellulose, hemicellulose, and lignin—significantly influence the product distribution. The abundant hydrogen free radicals generated from PP facilitate vigorous reactions with biomass-derived intermediates, thereby enhancing hydrocarbon formation. Additionally, lignin pyrolysis contributes to the generation of various oxygenated hydrocarbons, including ketones, aldehydes, furans, and phenols [16]. Research indicates that maximizing hydrocarbon yield can be achieved by selecting cellulose-rich, lignin-poor biomass feedstocks—such as corn stove—and co-pyrolyzing them with PP at 600 °C, a condition shown to significantly improve hydrocarbon production while minimizing catalyst coking. Conversely, for the targeted production of phenolic compounds, biomass with high lignin content—such as chestnut shells—is recommended, as such materials are particularly suitable for hydrothermal conversion into high-value phenolic chemicals. These insights provide a scientific basis for precise control over product selectivity and for optimizing the alignment between feedstock, process parameters, and desired output.

The co-thermal pyrolysis of lignocellulosic biomass (e.g., CS) with PP produces higher yields of hydrocarbon products, whereas the co-thermal pyrolysis of lignocellulosic residues (e.g., CT) with PP generates high-value phenolic compounds. In the actual process, cellulose is usually pre-extracted to obtain the residue rich in hemicellulose and lignin, and then the next step is carried out. The hydrogen supplied by PP helps stabilize lignin-derived phenolic oxygen radicals, prevents excessive carbonation, and maximizes the production of liquid chemicals from this low-value industrial waste [35]. The study also provides a feasible technical path for recycling such extracted cellulose waste.

### 3.4. GHG Emission and Techno-Economic Analysis

**Figure 5:**
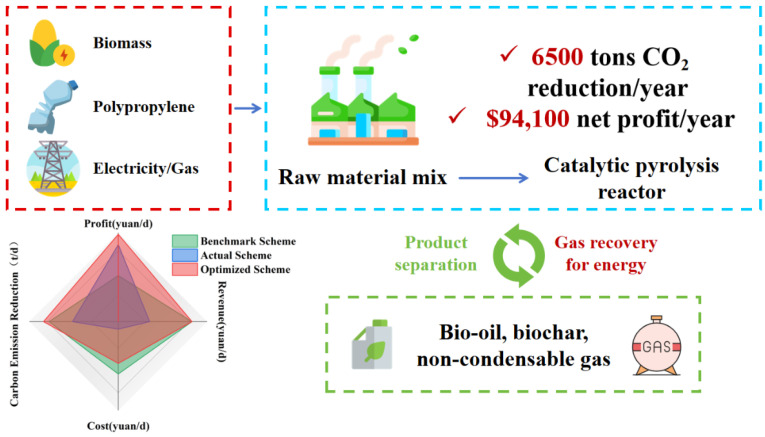
System boundary and benefit assessment framework for GHG calculation.

As illustrated in Figure 5, the pyrolysis process under Scenario A demonstrates a daily profit advantage of approximately 11,684 RMB relative to the economically non-viable baseline (Scenario B). Research has shown that pyrolysis technology can significantly enhance environmental sustainability by reducing greenhouse gas emissions, supporting carbon neutrality, and promoting resource recycling. For example, a study of fast pyrolysis of straw biomass revealed a marked reduction in greenhouse gas emissions compared to conventional open-field burning practices, as well as reduced pollution associated with waste treatment. Recent studies have shown that the integration of resources and carbon capture technologies can effectively quantify and manage annual net carbon dioxide emissions [54]. However, Scenario A generates approximately 6349 tons more CO_2_ emissions than Scenario B, suggesting limited carbon reduction performance and no additional emission reductions beyond the baseline level. In contrast, Scenario C—optimized based on engineering principles—demonstrates superior economic and environmental performance. By employing a hybrid energy supply system combining electricity and natural gas, Scenario C achieves a net daily profit increase of approximately 22,288 RMB over the baseline and realizes significant environmental benefits. Specifically, when processing 1200 tons of municipal solid waste per day, Scenario C reduces annual net CO_2_ emissions by approximately 155,478 tons—4917 tons more than the reduction achieved by Scenario B.

This assessment clearly demonstrates that directly scaling up laboratory conditions is not economically viable. However, through energy system optimization in Scenario C, the co-pyrolysis technology proposed in this study can not only overcome economic barriers but also achieve profitability and carbon reduction potential that far surpass those of the existing process (Scenario A). Therefore, the key to transitioning this technology from the laboratory to industrial-scale application lies in the integration and optimization of the energy system. By replacing the fully electric heating model with a more cost-effective hybrid energy solution, the substantial environmental benefits of this technology can be fully realized and translated into significant economic value, thereby providing a clear pathway for its commercial deployment.

## 4. Conclusions

This study systematically investigated the co-pyrolysis behavior of four typical biomass types (RH, PI, CS, and CT) with PP. The results demonstrate that incorporating PP into biomass co-pyrolysis systems significantly reduces the average activation energy, with reductions reaching up to 54.90% in specific cases. As an effective hydrogen donor, PP facilitates hydrogen transfer and free radical reactions, thereby lowering the reaction barrier and enhancing overall reaction activity during biomass pyrolysis. The distribution of pyrolysis products is highly dependent on temperature. At low temperatures (300–400 °C), the primary products are small-molecule alkanes, along with minor amounts of aromatic compounds. At medium to high temperatures (500–600 °C), dehydrogenation and chain scission reactions become dominant, leading to a substantial increase in the yield of medium-to high-molecular-weight hydrocarbons. However, at excessively high temperatures (700 °C), secondary cracking occurs, resulting in reduced yields of certain hydrocarbons due to decomposition. Although this study has made significant progress, there are still limitations that require further investigation. The waste of food packaging is highly heterogeneous and usually contains other main olefin-based plastics, so the mixed effect of other plastics must be considered in the practical application.

The intensity of the synergistic effect is closely associated with the chemical composition of the biomass. During the co-pyrolysis of cellulose-rich CS with PP, a pronounced synergistic effect significantly enhances hydrocarbon yield, increasing it from 39.8% in individual pyrolysis to 65.6%. In contrast, CT has a high lignin content, and exhibits a single mass loss peak and produces a higher proportion of phenolic compounds, thereby limiting hydrocarbon formation. Although an all-electric heating system may be theoretically optimal for environmental sustainability, its high capital and operational costs render it economically unfeasible. An optimized process utilizing natural gas as an energy source effectively balances economic viability with environmental performance, achieving a substantial daily net profit of 1835 RMB and the highest annual net CO_2_ emission reduction of 4917 tons. The co-pyrolysis of biomass and polypropylene not only enables the efficient and synergistic conversion of these waste streams into high-value products but also yields high-quality biofuels. Under the optimized configuration, this process demonstrates significant economic and environmental benefits, presenting a promising technological pathway toward sustainable energy development and the realization of the “dual carbon” goals.

## Figures and Tables

**Table 1:** Input–output data for GHG calculation.

Category	Items	Unit	Scenario A	Scenario B	Scenario C	Data Source
Inputs						
Raw Materials	Biomass/PP Mixture	kg	20,000	20,000	20,000	Measured
Energy	Electricity	kWh	5582	9357	1684	Metering/Calc.
	Natural Gas	Nm^3^	564	0	374	Calculated
Outputs						
Products	Pyrolysis Oil	kg	---	8100	11,600	Exp. Data
	Solid Char	kg	---	3240	4800	Exp. Data
	Syngas (Non-condensable)	kg	6000	6660	3600	Exp. Data
Waste	Solid Residue	kg	8000	10,660	5330	Plant Data

---: not detected.

**Table 2:** Physicochemical properties of biomass and polypropylene (PP).

	Physicochemical Property	RH	PI	CS	CT	PP
Elemental	C (%)	38.72 ± 0.94	46.15 ± 0.91	49.69 ± 0.36	72.67 ± 0.73	84.88 ± 0.53
	H (%)	4.96 ± 0.21	5.48 ± 0.16	5.66 ± 0.02	5.30 ± 0.09	12.53 ± 0.05
	O (%)	55.70 ± 0.42	47.55 ± 0.46	43.90 ± 0.38	16.65 ± 0.69	2.60 ± 0.03
	N (%)	0.61 ± 0.06	0.82 ± 0.08	0.76 ± 0.01	5.38 ± 0.05	0
Proximate	S (%)	0	0	0	0	0
	Moisture M_ad_ (%)	6.26 ± 0.08	6.16 ± 0.08	4.81 ± 0.35	5.62 ± 0.32	0.71 ± 0.02
	Ash M_ad_ (%)	0.95 ± 0.15	0.95 ± 0.15	5.82 ± 0.62	7.12 ± 0.35	0.00
	Volatile Matter M_ad_ (%)	63.05 ± 0.11	76.67 ± 0.11	78.12 ± 0.83	65.35 ± 0.38	99.29 ± 0.01
	Fixed Carbon M_ad_ (%)	15.61 ± 0.07	16.22 ± 0.07	11.25 ± 0.30	21.91 ± 0.68	0.00
Composition	Cellulose (%)	34.53 ± 0.08	41.00 ± 0.14	38.76 ± 0.05	20.55 ± 0.05	/
	Hemicellulose (%)	19.56 ± 1.61	14.48 ± 2.11	21.58 ± 0.35	17.42 ± 2.05	/
	Lignin (%)	26.27 ± 0.70	26.16 ± 0.56	17.50 ± 0.16	47.78 ± 0.65	/

## Data Availability

The original contributions presented in this study are included in the article. Further inquiries can be directed to the corresponding author.
